# Desmoplastic Small Round Cell Tumors: Clinical Presentation, Molecular Characterization, and Therapeutic Approach of Seven Patients

**DOI:** 10.1155/2024/5036102

**Published:** 2024-10-08

**Authors:** Verena I. Gaidzik, Regine Mayer-Steinacker, Mathias Wittau, Markus Schultheiß, Alexandra v. Baer, Kathrin Oehl-Huber, Sonja Dahlum, Anja Fischer, Uwe Gerstenmaier, Thomas Seufferlein, Andreas Buck, Ambros Beer, Wolfgang Thaiss, Peter Möller, Hartmut Döhner, Reiner Siebert, Ralf Marienfeld, Thomas F. E. Barth

**Affiliations:** ^1^Department of Internal Medicine III, University Hospital of Ulm, Ulm, Germany; ^2^Center of Personalised Medicine, University Hospital of Ulm, Ulm, Germany; ^3^Department of General and Visceral Surgery, University Hospital of Ulm, Ulm, Germany; ^4^Department of Trauma-, Hand- and Reconstructive Surgery, University Hospital of Ulm, Ulm, Germany; ^5^Institute of Human Genetics, University Hospital of Ulm and University of Ulm, Ulm, Germany; ^6^Institute of Pathology, University of Ulm, Ulm, Germany; ^7^Department of Internal Medicine I, University Hospital of Ulm, Ulm, Germany; ^8^Department of Nuclear Medicine, University Hospital of Würzburg, Würzburg, Germany; ^9^Department of Nuclear Medicine, University Hospital of Ulm, Ulm, Germany

## Abstract

Desmoplastic small round blue cell tumor (DSRCT) is a highly aggressive fatal sarcoma without evidence-based therapeutic guidelines. We present here seven patients with DSRCT including immunohistochemistry combined with fluorescence in situ hybridization (FISH), next generation sequencing (NGS, *n* = 6) as well as OncoScan array (*n* = 3) analyses and show consecutive therapeutic approaches. All seven DSRCT patients presented with an extended abdominal mass; median age at diagnosis was 24.8 years. NGS analyses revealed five class 4 or 5 sequence variants. Remarkably, OncoScan and targeted analyses by FISH identified genomic gains of *CCND1* in two cases. Cyclin D1 expression was present in all seven tumors as shown by immunohistochemical staining. Multimodal therapeutic concepts included systemic therapies, resection, and radiation. Six patients were treated as first-line therapy with conventional chemotherapy. All except one patient had a dismal therapy response. Subsequent therapy lines consisted of chemotherapeutic combinations followed by targeted therapies. Due to Cyclin D1 expression, the CDK4/6 inhibitor palbociclib was applied to four patients. The median therapy duration until disease progression in these patients was 4.5 months (range, 1.5–5 months). So, *CCND1* genomic gain and Cyclin D1 expression are common features pointing to cell-cycle deregulation as a possible therapeutic target.

## 1. Introduction

Desmoplastic small round blue cell tumor (DSRCT) is a rare highly aggressive soft tissue sarcoma composed of small round tumor cells showing a distinctive pattern of polyphenotypic differentiation surrounded by a prominent desmoplastic stroma [1–5]. Typically, DSRCT (ICD-O: 8806/3) is characterized by recurrent chromosomal translocation t(11;22)(p13;q22) resulting in fusion of the *EWS RNA Binding Protein 1 (EWSR1)* gene on 22q12.2 and the *Wilms Tumor 1 (WT1)* gene on 11p13 involving different genomic breakpoints [1–4, 6]. DSRCT predominantly affects children and young adults; the vast majority of DSRCT develops in the abdominal cavity, frequently in the retroperitoneum, pelvis, omentum, and mesenterium with multiple tumor nodules studding the peritoneal surface [1, 3, 6]. Further localizations have been described in very few cases, e.g., arising in the head and neck [4]. Clinical symptoms are related to the primary side of presentation and comprise pain, abdominal distension, acute abdomen, and organ obstruction. DSRCT are usually immunoreactive to cytokeratins, epithelial membrane antigen (EMA), WT1, and desmin. Detection of *EWSR1* rearrangement is a desirable diagnostic characteristic in cases with unusual clinical and histological features according to the current classification of the World Health Organization [1, 3, 6].

Current treatment strategies consist of multimodal therapeutic concepts including cytoreductive surgery, intensive chemotherapy, and involved field radiotherapy; for some patients, surgery has been combined with hyperthermic intraperitoneal chemotherapy [3–12]. Extended surgery has been described to be associated with improved survival in DSRCT patients [13]. In recent years, targeted therapeutic approaches have begun to emerge since the characteristic *EWSR1*::*WT1* rearrangement leads to down-stream activation of many signaling pathways known to be involved in carcinogenesis, e.g., *platelet-derived growth factor* (*PDGF*), *vascular endothelial growth factor* (*VEGF*), *insulin growth factor* (*IGF*)-1 pathways, or DNA damage repair [6, 11]. Drugs of interest are multityrosine kinase inhibitors (TKI) such as pazopanib, imatinib, and sorafenib alone or in combination with other agents such as mTOR (mammalian target of rapamycin) inhibitors [11]. Further deregulated pathways, e.g., mesenchymal-epithelial reverse transition (MErT)/epithelial-mesenchymal transition (EMT) and DNA damage repair support therapy rationale with PARP-inhibitors [6, 12, 13]. Despite these multimodality therapy efforts, the prognosis of DSRCT is still dismal with a 5-year overall survival rate of 10–15%, underlining the utmost need for further therapeutic options [6, 11].

We present here the data of seven patients with DSRCT treated at our institution, taken from clinical and molecular analyses, as well as clinical decisions resulting in therapeutic approaches.

## 2. Materials and Methods

### 2.1. Patient Samples and Collection

We identified seven patients with DSRCT who were treated between 2017 and 2019 in the Department of Internal Medicine III of the University Hospital of Ulm, Germany, and retrieved corresponding paraffin embedded tissue blocks from the files of the Institute of Pathology, University Hospital of Ulm, Germany.

The research was approved according to the local ethic committee of the University of Ulm (reference 369/17) and is in compliance with the ethical principles of the Declaration of Helsinki [14, 15].

### 2.2. Sample Analyses

In all seven patients, conventional histology and immunohistochemistry (IHC) were performed with the following diagnostic antibodies: vimentin (VIM3B4), desmin (DE-R-11), CD56 (123C3), and CK8/18 (all Agilent Dako, Glostrup, Denmark). In addition, IHC was applied for WT1 (C-terminus, Ab15249, Abcam, Cambridge, UK), PDGFR-1 (D13C6, Cell signaling, Cambridge UK), PD1 (JAD1, Dianova, Hamburg, Germany), p16 (1D7D2; Invitrogen, ThermoFisher Scientific, Waltham Massachusetts USA), CyclinD1 (EP12; Agilent Dako, Glostrup, Denmark), cyclin-dependent kinase 4 (CDK4; Clone QDCS-31.2, Zytomed, Berlin, Germany), cyclin-dependent kinase 6 (CDK6 clone 8H4, LS Bio Seattle Washington, USA), retinoblastoma protein (Rb, clone 4H1, 4H1; Cell Signaling, Cambridge, UK), and phosphorylated Rb (pRbSer780, 9307, Cell Signaling, Cambridge, UK). Fluorescence in situ hybridization (FISH) for *EWSR1* rearrangement was performed according to published protocols (Vysis LSI EWSR1 Dual Color Break Apart Rearrangement Probe, Ref 30–190059, Abbott, Abbott Park, IL, USA) [16].

For next generation tumor-only sequencing (NGS, *n* = 6), a 160-gene panel (Human Comprehensive Cancer Panel, Qiagen) was used. Library preparation was done using at least 40 ng of DNA according to the instructions of the manufacturer. Analyses were performed on a MiSeq device (Illumina) with a mean coverage of 2006-4257X (100X minimal coverage, range 92–99% of target regions). CLC Biomedical Workbench 5.0.1 (for patient 1) or CLC Genomic Workbench 12 (for patients 2–5 and 7) was used for variant calling. All variants are displayed in Supplemental Table 1 (annotated using ANNOVAR) [17]; only class four and five variants classified according to Richards et al. are reported in the results part [18].

OncoScan array analyses (Affymetrix, *n* = 3) were performed on the DNA of tumors and evaluated using the ChAS software. The copy number changes detected in the three tumors are shown in Supplemental Table 1. Focal imbalances of <5 Mb were considered as indicators of potential therapy targets.

## 3. Results

### 3.1. Clinical Characteristics

Overall, there were six male patients and one female with a median age of 24.8 years at the time of diagnosis (range, 21–34 years of age; Table 1). The DSRCT presented in all patients with abdominal pain due to an extended abdominal mass accompanied by peritoneal spreading. In two patients, histologic diagnosis was performed on samples from a cervical lymph node. During the course of disease, all patients suffered from distant metastases. One patient had bone metastases.

### 3.2. Comprehensive Pathologic and Molecular Genetic Approach

**Figure 1:**
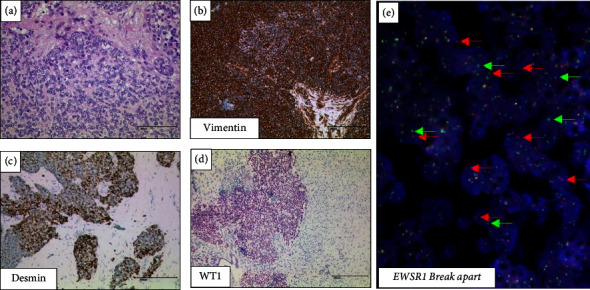
Histology and immunohistochemistry as well as fluorescence in situ hybridization from the desmoplastic small round cell tumor of patient 1. Hematoxylin and eosin staining (a), immunohistochemistry for vimentin (b), desmin (c), WT1 C terminus (d), and multiple single red and green signals in the break-apart FISH assay indicative of an *EWSR1* rearrangement in hyperdiploid tumor cells (e); bars, 200 *μ*m.

All tissue samples revealed a typical histology with sharply outlined nests of round neoplastic cells within a prominent desmoplastic stroma and characteristic (immuno-) histological profile showing positivity for vimentin, desmin (dot-like), WT1 C-terminus, CD56, and cytokeratin. Consistently, we detected a rearrangement of *EWSR1* indicative of a t(11;22)(p13;q12) in all seven patient samples (see Figure 1 and Table 1).

To identify potential molecular therapeutic targets, we conducted NGS analyses in six of seven patient samples. All identified variants are displayed in Supplemental Table 1. With regard to (likely) pathogenic variants (Table 1), one patient was found to have a sequence variant in *HNF1A* (p.P291fs, variant allele frequency [VAF] 4.5%) and two patients with a sequence variant in *KIT* (p.M541L, VAF 6.9% and 4.1%). One patient revealed sequence variants in *KIT* (p.M541L, VAF 34.4%) and *PIK3CA* (p.Y1021C, VAF 25.5%). There were no class 4 or 5 mutations in the other two patients.

**Figure 2:**
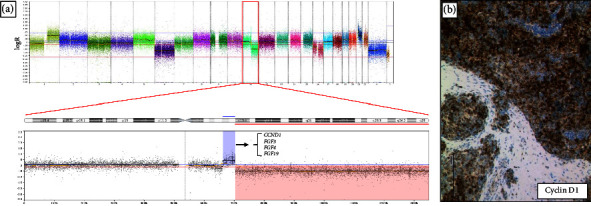
OncoScan Array analyses of patient 1 reveal a peak-like signal indicative of a genomic gain in the array analysis on chromosome 11 including *CCND1* gene (a); immunohistochemistry for Cyclin D1 in the tumor of the same patient (b); bar, 200 *μ*m.

OncoScan analyses were performed in three patients and revealed a focal amplification in 11q13.2-q13.3 encompassing the *CCND1* gene with coamplification of *FGF19*, *FGF4*, and *FGF3* in one case (Supplement Table 1); combined with subsequent targeted analyses by FISH, we identified a genomic gain of the *CCND1* locus in two cases (Figure 2(a)). Corroborating and extending this genomic finding, Cyclin D1 protein expression was detected in all seven tumors (Figure 2(b)). In addition, in one patient, OncoScan analyses revealed a copy number gain of chromosome 5 containing the genes *FGF1*, *PDGFRB*, and *FGFR4* known as regulators in DSRCT (Table 1) [9].

### 3.3. Multimodal Therapies

**Figure 3:**
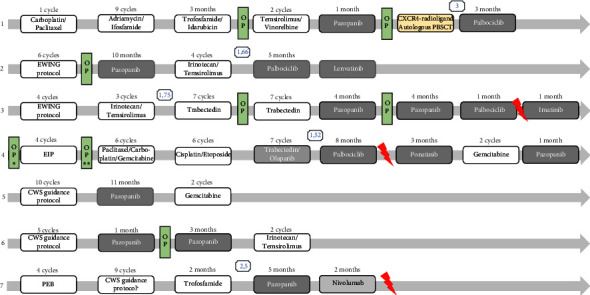
Graphic overview of applied multimodal therapies in all seven patients with desmoplastic small round cell tumor. 1–7, number of patients; OP, surgical resection; R, radiotherapy. Comparison of progression-free survival (PFS) achieved by a new treatment (PFS2) to the PFS of the most recent treatment on which the patient has experienced progression (PFS1) with a defined clinical benefit as a PFS ratio (PFS2/PFS1) >1.3 was added (blue number): patient 1: 3; patient 2: 1.66; patient 3: 1.75; patient 4: 1.52; patient 7: 2.5.

A median of five systemic therapy lines were applied to the seven DSCRT patients (range, 3–8; Figure 3). Six out of seven patients were treated with conventional chemotherapy as first-line therapy with the following regimens: 1 cycle carboplatin/paclitaxel (*n* = 1), CWS guidance protocol (10 cycles, *n* = 1; 5 cycles, *n* = 1), EWING protocol (6 cycles, *n* = 1; 4 cycles, *n* = 1), and PEB (4 cycles, *n* = 1). One patient received adjuvant chemotherapy with the EIP regimen (4 cycles, *n* = 1) after initial abdominal tumor resection (see also Supplement Table 2). All except one patient had a dismal therapy response with three partial remissions (PR) and three stable diseases (SD) after first-line therapy. One patient achieved into complete remission (CR) with a duration of two years. Second and further therapy lines consisted of different chemotherapeutic combinations (ifosfamide/adriamycin or actinomycin, *n* = 1; irinotecan/temsirolimus, *n* = 3; CWS guidance protocol, *n* = 1; paclitaxel/gemcitabine/carboplatin, *n* = 1; trofosfamide/idarubicin, *n* = 1; temsirolimus/vinorelbine, *n* = 1; trofosfamide, *n* = 1; trabectedin, *n* = 1; cisplatin/etoposide, *n* = 1; gemcitabine, *n* = 2) as well as biosimilars (pazopanib, *n* = 7; olaparib [with trabectedin], *n* = 1; palbociclib, *n* = 4; imatinib, *n* = 1; lenvatinib, *n* = 1). One patient received immune checkpoint inhibitor therapy with nivolumab. Five patients underwent abdominal tumor resection; of these, three patients received surgery twice during their course of disease. Three patients were treated with palliative radiotherapy. All seven patients with DSRCT received therapy with pazopanib with a median time on therapy of 4 months (range, 1–11 months), in three of seven patients as second-line therapy.

Since Cyclin D1 was expressed in all patients, we were interested whether other key regulators of cell cycle pathways were deregulated. Therefore, we performed immunohistochemistry for p16 (also known as cyclin-dependent kinase inhibitor 2A, CDKN2A), CDK4 (cyclin-dependent kinase 4), CDK6 (cyclin-dependent kinase 6), Rb (retinoblastoma protein), and pRB (phosphorylated Rb). All tumor samples revealed positivity for CDK4, CDK6 (except in one patient), Rb, and pRb, reinforcing cell cycle deregulation (Supplement Figure 1). After these results had been discussed in our molecular and familial tumor board and reimbursement of treatment costs by the statutory health insurance upon request targeted therapy with the CDK4/6 inhibitor, palbociclib was applied to four patients (1, 2, 3, 4); these patients were informed about the off-label use as well as potential side effects and gave their written informed consent for this therapy. The four patients treated with palbociclib had a median response to therapy of four months (range, 1–8 months). Therapy was terminated due to progressive disease (PD, *n* = 3) and one mixed response (*n* = 1) without relevant side effects. In the other three patients, therapy with palbociclib was not feasible due to expected toxicity or due to rapid PD.

**Figure 4:**
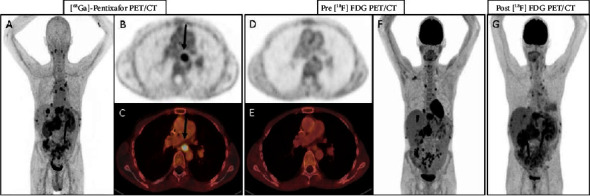
(A–C): [68Ga]Pentixafor PET/CT, (D–F), [18F]FDG PET/CT before application and (G) after application of therapeutic CXCR4 ligand, 2 GBq [90Y]Pentixather of patient 1.

We compared progression-free survival (PFS) achieved by a new treatment (PFS2) to the PFS of the most recent treatment on which the patient has experienced progression (PFS1) with a defined clinical benefit as a PFS-ratio (PFS2/PFS1) >1.3 [19]. A PFS2/PFS1 ratio >1.3 was achieved in three patients with palbociclib (patient 1 : 3; patient 2 : 1.66; patient 4 : 1.52) and in two patients with trabectedin (patient 3 : 1.75) or pazopanib (patient 7 : 2.5; Figure 3), respectively. In addition, we performed a [68GA]Pentixafor PET/CT scan with CXCR4-directed radioligand in one patient to evaluate the CXCR4 expression in the tumor aiming at a potential CXCR4-radioligand therapy (Figure 4). As the CXCR4 expression in the tumor was higher than in the normal tissues, the patient received a therapy combination of 8.2 GBq [90Y]Pentixather, a therapeutic CXCR4 ligand, and high-dose chemotherapy followed by autologous peripheral stem cell transplantation (University Hospital Würzburg). The control [18F]PET/CT revealed PR, and the patient received palbociclib as maintenance therapy until death by PD.

## 4. Discussion

DSRCT is a rare soft tissue sarcoma with a dismal outcome and limited therapeutic options [4, 6, 13, 20]. Here, we report a case series of seven patients including (immune-) histological analyses as well as molecular genetic approaches in order to identify targetable structures.

Predominantly, DSCRT are diagnosed in children and young adults which is in line with the age at diagnosis of our seven DSRCT patients ranging between 21 and 34 years of age. A common clinical feature in all reports and in our patients is an advanced abdominal tumor mass; in addition, all patients suffered from metastases during their course of disease [4, 6, 13, 20].

DSRCT is characterized by desmin-positive small round tumor cells, surrounded by desmoplastic connective/fibrous tissue which we also observed in our patient samples [3–5, 8, 9]. This desmoplastic tissue is discussed as being one of the reasons why these tumors do not respond to conventional therapies [7].

In a series of 60 patients <40 years of age treated in prospective CWS trials between 1997 and 2015, Scheer et al. described a longer event-free survival (EFS) of 29.4 months in 15 patients treated with the regimen VAIA (vincristine, dactinomycin D, ifosfamide, and doxorubicin) compared to other chemotherapeutic regimens [21]. Two of our patients were treated with ten and five cycles, respectively, as first-line therapy according to the CWS guidance protocol and a third patient with nine cycles as second-line therapy. In addition, DSCRT has been shown to be nonimmunogenic, thus further limiting new therapeutic options, e.g., immune checkpoint inhibition [22, 23]. This is underlined by novel data showing a downregulation of pathways including immune system function and focal adhesion. [6] Nevertheless, one patient revealed PD-1 expression in his tumor, providing the rationale for therapy with nivolumab. However, the condition of the patient deteriorated rapidly within one month under this therapy, and palliative radiotherapy for symptom control was initiated before the patient died due to PD.

A highly characteristic feature of DSRCT is chromosomal translocation t(11;22)(p13;q12) involving the activating domain of *EWSR1* and the tumor suppressor gene *WT1* [19]. Downstream activation caused by this gene fusion targets many “blockbuster” genes such as *PDGF, VEGF, FGFR*, or *KIT* known to be deregulated in many cancer types [9, 13, 22]. Based on this background and existing clinical data, the use of TKI has been established in DSRCT patients with relapsed or advanced disease. In these patients, SD was described rather than disease regression [12]. We observed SD with a median of five months during therapy with TKI pazopanib; in two of these patients, the therapy was used to improve the general condition of the patients, aiming at multivisceral resection. After surgery and completed wound healing, we continued therapy with pazopanib for three and four months, respectively. Interestingly, PDGFR expression in the IHC analysis did not correlate with the therapy response to pazopanib in these patients, suggesting another pathway deregulation. In the meta-analysis of Mello et al., different trials with pazopanib in sarcoma patients were analyzed focusing on the outcome of DSCRT patients. In this study, only one patient achieved CR; clinical benefits (PR and SD) ranged between 22% and 62% of the patients [13].

By applying NGS, we were able to detect isolated variants only in four patients, and among these, only two variants were potentially druggable. These were variants in *KIT* and *PIK3CA*. In line with the case report of de Sanctis and colleagues, therapy with imatinib in one patient showed only limited activity with a short time period of one month until PD; interestingly, this patient had a *KIT* mutation [24]. The PI3K-inhibitor alpelisib was not yet available for our patient.

There are known further activated and potentially targetable genes in DSRCT such as *ERK1/2, p38, SAPK/JNK*, and *AKT* [12, 13]. We combined chemotherapy (vinorelbine, *n* = 1) or irinotecan (*n* = 3) with the mTOR inhibitor temsirolimus in order to target the PI3K/AKT/mTOR pathway [12, 13]. Two patients received four and two cycles with vinorelbine/temsirolimus and another patient two cycles with vinorelbine/temsirolimus, respectively. CK4/6 inhibitors are approved for the treatment of breast cancer and are currently being investigated in different malignant neoplasms, e.g., chordomas. It was shown that chordoma cells are characterized by a frequent loss of *CDKN2A*, resulting in activation of the CDK4/6 and RB pathways as shown by the expression of CDK4/6/pRb (S780) [25]. In our cohort of DSRCT, we observed a gain/amplification of Cyclin D1, which is part of the CDK4/6/pRb (S780) pathway, in two patients. In addition, all seven patients showed Cyclin D1 expression in their tumor samples. Therefore, we initiated therapy with the CDK4/6 inhibitor palbociclib in four patients. Interestingly, the median therapy duration until disease progression in these heavily pretreated patients was 4.5 months. Three patients treated with palbociclib showed also a PFS2/PFS2 ratio >1.3. In an alternative approach, the novel CDK4/6 inhibitor ribociclib also showed effects in combination with goserelin and letrozole in a patient with multiple desmoid tumors [26]. In 5 of 68 (7%) of tumor samples, novel whole-exome sequencing data identified alterations in the *fibroblast growth factor receptor 4* (*FGFR4*) as a potential therapeutic target based on high expression, recurrent amplification, and recurrent activating mutations [27]. This is in line with our data, as we also detected a gain of chromosome 5 containing the *FGFR4* gene in the tumor of one patient by OncoScan analysis. Recent RNA-sequencing data compared primary and recurrent DSRCT samples as well as cell lines suggesting the *EWSR1::WT1* fusion protein as a principal driver with enrichment in genes regulated by the fusion protein in the recurrent versus primary tumor [6].

The CXC-chemokine receptor-4 (CXCR4) pathway plays a role in cancer cell homing and metastasis, representing a potential target for cancer therapy, i.e., CXCR4 activates mTOR and the Bruton tyrosine kinase [28]. [68Ga]Pentixafor is a radiolabeled CXCR4 ligand that allows noninvasive whole body PET imaging in multiple malignancies and inflammatory disease conditions [29]. In one of our patients, CXCR4 expression was detected by [68Ga]Pentixafor-PET/CT. As CXCR4 is physiologically expressed on hematopoietic stem cells, a CXCR4 radioligand therapy can be applied only in the case of sufficient stem cell support. In addition, high-dose chemotherapy before autologous stem cell transplantation is necessary. After this specific therapy, we treated our patient with palbociclib as maintenance therapy for three months before he died due to PD. However, this might be an interesting therapeutic strategy in selected centers and needs further investigation in DSRCT.

## 5. Conclusions

We describe a cohort of seven patients with DSRCT who were treated with different multimodal therapeutic approaches, ranging from tumor mass reduction, to palliative radiotherapy and various systemic therapies including intensive chemotherapies and targeted or molecular-guided therapies, respectively. Although the success of the treatment was limited, we showed that the application of novel targeted therapies could at least partially stabilize the disease with acceptable side effects even in heavily pretreated patients. Therefore, we suggest that therapy of patients with DSRCT be based on several adapted consecutive therapy lines. Nevertheless, further investigations are warranted to elucidate the hallmarks of DSRCT as a basis for more molecular-guided therapies in this rare tumor entity with a fatal clinical course.

Overview of clinical as well as pathologic and molecular genetic characteristics of all seven patients with desmoplastic small round cell tumors (DSRCT). The corresponding IHC is displayed in Supplement Figure 1.

## Figures and Tables

**Table 1:** Overview of clinical as well as pathologic and molecular genetic characteristics of all seven patients with desmoplastic small round cell tumors (DSRCT). The corresponding IHC is displayed in Supplement Figure 1.

Patient	1	2	3	4	5	6	7

Gender	Male	Male	Male	Male	Female	Male	Male

Age (at diagnosis in years)	34	31	24	21	33	29	26
Tumor localisation (at diagnosis)	Abdominal tumor, peritoneal carcinosis	Abdominal tumor, lymph node	Abdominal tumor, lymph node	Abdominal tumor	Abdominal tumor, peritoneal carcinosis	Abdominal tumor	Abdominal tumor, lymph node, liver, bone

Histology	Desmoplastic small round blue cell tumor						

Immunohistology							
Cyclin D1	+	+	+	+	+	+	+
p16	+/−	−	−	n.e.	+	10% +	5% +
CDK4	+	+	+	+	+	+	+
CDK6	+	+	+	5% +	+	+	+/−
Rb	+	+	+	5% +	+	+	+
pRb	n.e.	+	5% +	2% +	+	+	5% +

FISH	*EWSR1* rearrangement						

NGS Sequence variants	No class 4 or 5 mutation detected	*HNF1A* (p.P291fs)	*KIT* (p.M541L)	No class 4 or 5 mutation detected	*KIT* (p.M541L), *PIK3CA* (p.Y1021C)	*KIT* (p.M541L)	Not available

OncoScan ^∗^	*CN gain* 1q21.1-q44 11q13.2-q13.3 15q25.2 21q11.2-q22.3 22q12.2 *CN loss* 2q23.1 6p25.3-q27 11q13.3-q25 16p13.3-q24.3 *LOH* 12p13.33-q24.33 17p13.3-q22 21q21.2-q22.3	*CN gain* 5p15.33-q.35.3 7p22.3-q36.3 18p11.32-q23 22q11.1-q13.33 *CN loss* 1p36.11-p.35.3 6q14.3-q26 8q11.21-q11.21 13q11-q34			*CN gain* 1q32.1-q44‘ 14q32.33 Xp22.33 *CN loss* 3q12-q22‘		

+, all tumor cells positive; -, all tumor cells negative; +/-, 50% of tumor cells positive; percentages indicate positive cells in a subpopulation of the tumor.^∗^Subclonal copy number variants and changes in ploidy not reported; ‘: low quality, also seen in the second sample; n.e., not evaluable.

## Data Availability

Data are unavailable due to privacy or ethical restrictions.

## Supplementary Materials

Supplementary Materials Supplement Table 1. Genomic Copy Number Variations detected by OncoScan Analyses using the ChAS Software. Supplement Table 2. Chemotherapy protocols and respective chemotherapeutic drugs applied in the patient cohort. Supplement Figure 1. Immunohistology of a desmoplastic small round cell tumor (DSCRT) positive for CDK4, CDK6, retinoblastoma, protein, and phospho retinoblastoma protein.
