# Supplementary material for: Desmoplastic Small Round Cell Tumors: Clinical Presentation, Molecular Characterization, and Therapeutic Approach of Seven Patients
Source: Sarcoma. 2024 Oct 8;2024:5036102. doi: 10.1155/2024/5036102 (PMC11479773; doi:10.1155/2024/5036102)
Supplement: Supplementary Materials — Supplement Table 1. Genomic Copy Number Variations detected by OncoScan Analyses using the ChAS Software. Supplement Table 2. Chemotherapy protocols and respective chemotherapeutic drugs applied in the patient cohort. Supplement Figure 1. Immunohistology of a desmoplastic small round cell tumor (DSCRT) positive for CDK4, CDK6, retinoblastoma, protein, and phospho retinoblastoma protein. [file 5036102.f1.zip › Gaidzik_DSRCT_ Supplement Table 1.pdf]

Supplement Table 1: Genomic Copy Number Variations detected by OncoScan Analyses using the ChAS Software.

Patient	Type	Chromosomal region (hg19)	Cytoband	Length [kbp]	Selected genes
1	CN Gain	chr1:146,124,245-249,250,621	q21.1 - q44	103126377	
1	CN Loss	chr2:148,822,108-149,159,068	q23.1	336961	
1	CN Loss	chr6:1-171,115,067	p25.3 - q27	171115067	
1	CN Gain	chr11:66,263,828-70,316,602	q13.2 - q13.3	4052775	CCND1, FGF19, FGF4, FGF3
1	CN Loss	chr11:70,316,602-135,006,516	q13.3 - q25	64689915	
1	LOH	chr12:1-133,851,895	p13.33 - q24.33	133851895	
1	CN Gain	chr15:83,214,793-84,343,483	q25.2	1128691	
1	CN Loss	chr16:1-90,354,753	p13.3 - q24.3	90354753	
1	LOH	chr17:45,744-53,931,917	p13.3 - q22	53886174	
1	High Copy Gain	chr21:14,344,537-19,511,217	q11.2 - q21.1	5166681	
1	CN Gain	chr21:19,511,217-48,129,895	q21.1 - q22.3	28618679	
1	LOH	chr21:25,225,680-48,129,895	q21.2 - q22.3	22904216	
1	High Copy Gain	chr22:30,977,355-31,875,318	q12.2	897964	PIK3IP1
2	CN Gain*	chr1:202,687,486-242,996,817	q32.1 - q43	40309332	
2	CN Gain*	chr1:243,725,004-249,212,878	q44	5487875	
2	CN Loss*	chr3:98,788,651-99,422,587	q12.1	633937	
2	CN Loss*	chr3:100,295,733-123,188,936	q12.2 - q21.1	22893204	
2	CN Loss*	chr3:125,441,494-130,522,797	q21.2 - q22.1	5081304	
2	CN Loss*	chr3:131,838,077-132,149,664	q22.1	311588	
2	CN Loss*	chr3:133,485,133-133,682,278	q22.1	197146	
2	CN Gain	chr14:106,572,891-106,779,252	q32.33	206	
2	CN Gain	chrX:978,067-1,402,841	p22.33	425	
3	CN Loss	chr1:23952364-28347491	p36.11-p35.3	4395127	
3	CN Gain	chr5:38138-180698312	p15.33-q35.3	180660174	FGF1, PDGFRB, FGFR4
3	CN Loss	chr6:86816436-161725801	q14.3-q26	74909365	
3	CN Gain	chr7:41420-159118443	p22.3-q36.3	159077023	
3	CN Loss	chr8:48799663-48904099	q11.21-q11.21	104436	
3	CN Loss	chr13:19084822-115103150	q11-q34	96018328	
3	CN Gain	chr18:12841-78007784	p11.32-q23	77994943	
3	CN Gain	chr22:16054712-51213826	q11.1-q13.33	35159114	

* low quality, also seen in second sample

Footnote: Potential subclonal aberrations as well as changes of ploidy are not reported
