# Supplementary material for: Desmoplastic Small Round Cell Tumors: Clinical Presentation, Molecular Characterization, and Therapeutic Approach of Seven Patients
Source: Sarcoma. 2024 Oct 8;2024:5036102. doi: 10.1155/2024/5036102 (PMC11479773; doi:10.1155/2024/5036102)
Supplement: Supplementary Materials — Supplement Table 1. Genomic Copy Number Variations detected by OncoScan Analyses using the ChAS Software. Supplement Table 2. Chemotherapy protocols and respective chemotherapeutic drugs applied in the patient cohort. Supplement Figure 1. Immunohistology of a desmoplastic small round cell tumor (DSCRT) positive for CDK4, CDK6, retinoblastoma, protein, and phospho retinoblastoma protein. [file 5036102.f1.zip › Gaidzik_DSRCT_Supplement_Figure 1.pdf]

Supplement Figure 1

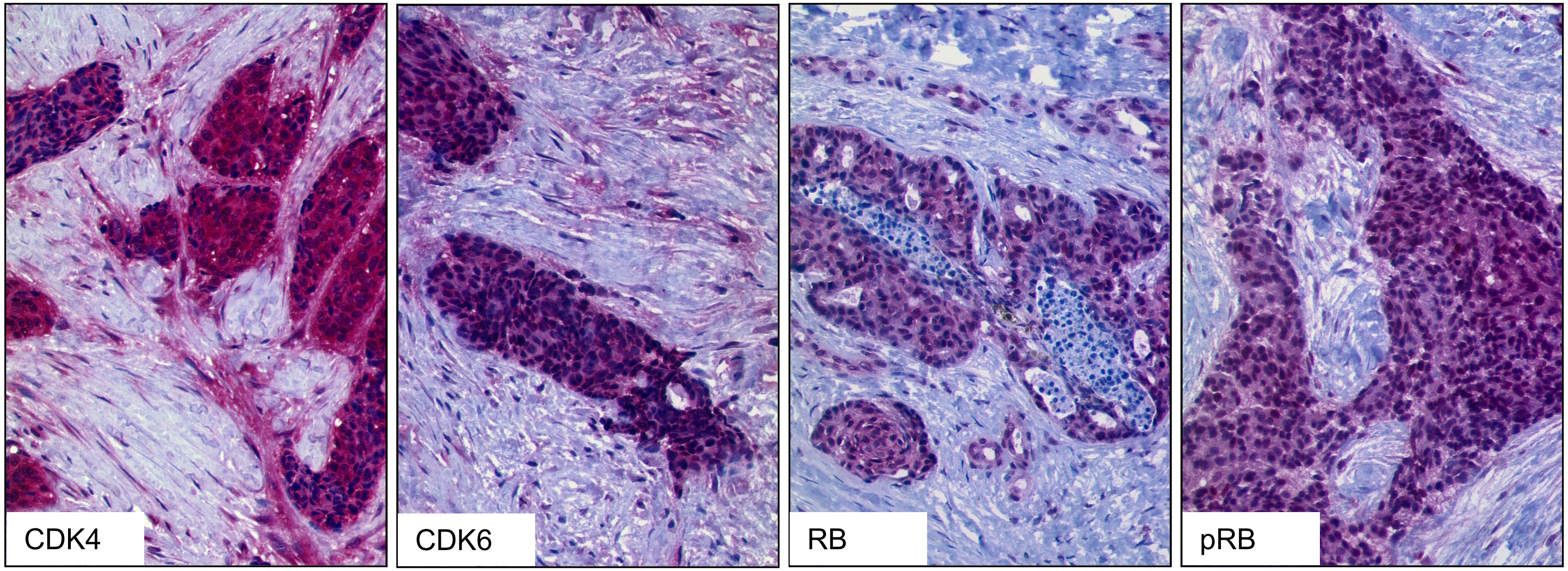

Immunohistology of a desmoplastic small round cell tumor (DSCRT) positive for CDK4, CDK6, retinoblastoma, protein and phospho retinoblastoma protein.
